# Polarization Smoothing Generalized MUSIC Algorithm with Polarization Sensitive Array for Low Angle Estimation

**DOI:** 10.3390/s18051534

**Published:** 2018-05-12

**Authors:** Jun Tan, Zaiping Nie

**Affiliations:** School of Electronic Science and Engineering (National Exemplary School of Microelectronics), University of Electronic Science and Technology of China, Shahe Campus, Section 2, North Jianshe Road, Chengdu 610054, China; zpnie@uestc.edu.cn

**Keywords:** DOA, VHF, multipath, PSA, low angle estimation, polarization smoothing

## Abstract

Direction of Arrival (DOA) estimation of low-altitude targets is difficult due to the multipath coherent interference from the ground reflection image of the targets, especially for very high frequency (VHF) radars, which have antennae that are severely restricted in terms of aperture and height. The polarization smoothing generalized multiple signal classification (MUSIC) algorithm, which combines polarization smoothing and generalized MUSIC algorithm for polarization sensitive arrays (PSAs), was proposed to solve this problem in this paper. Firstly, the polarization smoothing pre-processing was exploited to eliminate the coherence between the direct and the specular signals. Secondly, we constructed the generalized MUSIC algorithm for low angle estimation. Finally, based on the geometry information of the symmetry multipath model, the proposed algorithm was introduced to convert the two-dimensional searching into one-dimensional searching, thus reducing the computational burden. Numerical results were provided to verify the effectiveness of the proposed method, showing that the proposed algorithm has significantly improved angle estimation performance in the low-angle area compared with the available methods, especially when the grazing angle is near zero.

## 1. Introduction

Meter wave radar is a direct and effective anti-stealth measure because the meter wavelength is located in the resonance band for most types of aircrafts, with the shape stealth technology being no longer valid in this band. Moreover, the wave absorbing efficiency of the dielectric coating is remarkably corrupted in this band [1]. However, Direction of Arrival (DOA) estimation in low-angle areas is still one of the most challenging problems, especially for the Very High Frequency (VHF) radar system, which has antennae that are severely restricted in terms of aperture and height. The poor performance of the angle estimation and the height measuring, especially in the low-angle tracking area, limits the application of meter-wave radars. The cause of this problem is the existence of the multipath effect. In low-angle estimation for the VHF radar, the direct signal and multipath signal lying in the main lobe are coherent and therefore, it is difficult to distinguish them in the spatial, temporal and Doppler domains.

Many efforts have been exerted in the recent decades to overcome the multipath effect. These efforts can be mainly divided into two broad categories: the extended monopulse methods [2,3,4,5,6,7] and the array signal processing techniques [8,9,10,11,12,13,14,15,16,17,18]. The former includes the complex indicated angle method, the double-null method and the fixed-beam method [4,5]. Although these methods have low computational complexity, the accuracy of the solution is reduced when the elevation angle of the incident wave is less than one-fourth of the beam [3]. In [6,7], iterative processing was used to estimate the elevation angle based on monopulse techniques, calculating the reflection angle and updating the null position in beamforming to eliminate the multipath effect. However, these methods heavily depend on the electrical size of the antenna aperture, which is not suitable for meter-wave radar. The array signal processing methods are featured frequently in studies, such as Maximum Likehood (ML) approaches [8,12,16,18], multiple signal classification (MUSIC) algorithms [10,11,16,17] and so on. The ML method requires statistical information about the signals and its computational cost dramatically increases with an increase in the number of searching angles. The Refined Maximum Likehood [8] (RML) method is based on a high determined multipath model, which is not always available in practices. The MUSIC algorithms use the subspace decomposition to differentiate between the direct and specular signals, which requires preprocessing to eliminate the coherence between the direct and specular signal for low-angle estimation problems. There are three main approaches to eliminate the coherence. First, there is the spatial smoothing method [10,11], which may reduce the effective array aperture length and result in lower resolution and accuracy. Second, there is the frequency diversity method [19], which is based on the phase difference between the direct and the specular signals that varies with frequency. It is easily available with the X-band radar. Third, there is the polarization smoothing method [16,17]. This approach is based on the phase difference between the direct and the specular signals for different polarization. Unlike the spatial smoothing methods, the polarization smoothing method is not limited to a specific array geometry and it does not decrease the effective of array apertures [16]. Furthermore, it does not require a wide bandwidth of frequency. In addition, a previous study [20] indicated that the polarization diversity strategy has better performance than the frequency diversity method for low-angle estimation problem, even when using a smaller number of snapshots.

The polarization smoothing technique is based on the Polarization Sensitive Array (PSA). The PSA is exploited to resolve the low-angle estimation problem in this paper. The vector sensitive array was proposed for source localization by Nehorai [21]. Rahamim proposed polarization-smoothing algorithm [16] in the vector sensitive array under multipath conditions to decorrelate the coherent signals. Hurtado and Nehorai analyzed the Cramer-Rao Bound (CRB) of the vector sensitive array for low-angle tracking problems [22], which indicated that the vector sensitive array has better performance than the traditional phase array. Xu developed the Polarization Smoothing MUSIC (PS-MUSIC) algorithm, which combines the Polarization Smoothing pre-processing and the MUSIC algorithm for low-angle tracking in VHF polarization sensitive radars [17]. This approach is based on the phase difference of the reflected signals between the vertical and horizontal polarization. However, when the grazing angle is close to zero, there is very little difference in the reflection coefficient between the horizontal and vertical polarization, which leads to poor decorrelation performance for the polarization smoothing processing and poor low angle estimation performance. It can utilize spatial smoothing [23] and polarization smoothing pre-processing simultaneously to improve the decorrelation performance, which could improve the low-angle estimation performance. The Polarization Smoothing and forward/backward Spatial Smoothing MUSIC (PS-SS-MUSIC) algorithm was discussed in a previous study [16] for vector sensor arrays. However, the spatial smoothing procedure may cause the aperture loss as a trade-off, which could decrease the angle estimation accuracy. Although the polarization smoothing algorithm can be adapted to the multiple-input multiple-output (MIMO) radar system for coherent sources [24,25], the polarization smoothing algorithm cannot be directly utilized in the MIMO radar system for low-angle estimation due to the coupling term in the multipath signal [26].

The Generalized MUSIC algorithm (GMUSIC) [27] can be used to solve the low-angle estimation problem using the composite searching vector instead of ordinarily searching vector, which does not need to solve the coherent problem. However, the GMUSIC algorithm is unable to be applied when the phase of the fading coefficient is $\pm 180^{{}^{\circ}}$ for the traditional phase array. A PSA that contains the horizontal and vertical polarized components can fix that problem by using the different phases of the fading coefficient between the horizontal and vertical polarization. In order to improve the low-angle estimation performance, especially when the grazing angle is close to zero, the Polarization Smoothing Generalized MUSIC (PS-GMUSIC) algorithm is proposed in this paper. The proposed algorithm exploits the composite searching vector instead of ordinary searching vector, making it unnecessary to solve the coherent problem. Furthermore, the proposed method based on the PSA does not have limitations in terms of the phase differences between the direct and the reflected signals. In addition, the geometry information of the symmetry multipath model is exploited to transform the two-dimensional angle search into one-dimensional angle search in order to reduce the computational burden for the proposed algorithm. The simulation results indicated that the proposed method has better low-angle estimation performance than the PS-MUSIC and PS-SS-MUSIC algorithms.

The rest of the paper is organized as follows: the multipath signal model of the polarization sensitive array and the derivation of CRB are described in Section 2. The polarization smoothing Generalized MUSIC algorithm (PS-GMUSIC) is proposed in Section 3. The simulation results and discussions are presented in Section 4. Finally, Section 5 provides our conclusions.

## 2. Multipath Model for Polarization Sensitive Array

**Figure 1:**
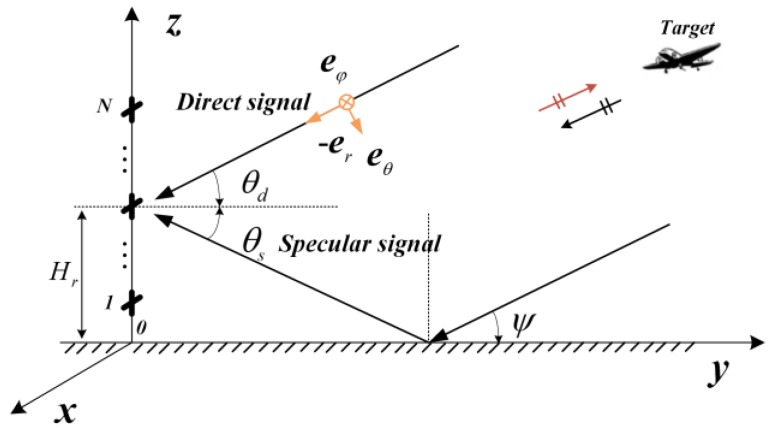
Multipath Model for Polarization Sensitive Array.

The multipath echoes are comprised of a single specular reflection and diffuse reflections. For a rough surface, the diffuse component dominates, while the reflected signals consist mainly of the specular component for a smooth surface. The ground can be considered as a smooth surface at the VHF band, because the Rayleigh roughness criterion can be easily satisfied due to the long wavelength based on the Rayleigh roughness criterion. Thus, the diffuse reflection components are ignored and the ‘double-ray’ symmetry multipath model is used, which is shown in Figure 1.

As shown in Figure 1, the N Polarization Sensitive Array (PSA) elements are uniformly distributed on the *Z*-axis, while the orthogonal electric dipoles are arranged along the *X*- and *Z*-directions. The distance between elements is equal to a half-wavelength. In this figure, $H_{r}$ is the height of the PSA; $\theta_{d}$ is the elevation angle of the direct signal of the target; $\theta_{s}$ is the depression angle of the specular signal; and ψ is the grazing angle of the target. According to the symmetry geometrical information of the symmetry multipath model (Figure 1), we can obtain that:

(1) $$\{\begin{array}{c} \theta_{s}=-\theta_{d}\\ \psi =\theta_{d} \end{array}$$

Supposing the echo is a completely polarized wave as shown in the Figure 1, the direct electric field vector can be expressed on the polarization basis (θ,φ) as:

(2) $$e_{d}=E_{\varphi }e_{\varphi }+E_{\theta }e_{\theta }=cos\gamma e_{\varphi }+sin\gamma e^{j\eta }e_{\theta },$$

where γ is the polarization angle; η is the phase difference of the different polarized components; φ and θ are the unit vectors in the Spherical Coordinates with $e_{\varphi }={\left[-sin\varphi ,cos\varphi ,0\right]}^{T}$ and $e_{\theta }={\left[sin\theta cos\varphi ,sin\theta sin\varphi ,-cos\theta \right]}^{T}$; and $E_{\varphi }=cos\gamma$ and $E_{\theta }=sin\gamma e^{j\eta }$ are the electric field amplitude of the unit vectors $e_{\varphi }$ and $e_{\theta },$ respectively. Obviously, although there are three components of the completely polarized electric field, only two of them are independent. Hence, we assumed that φ=π/2 in this paper as shown in Figure 1. The unit vectors of $e_{\varphi }$ and $e_{\theta }$ can be simplified as $e_{\varphi }={\left[-1,0,0\right]}^{T}$ and $e_{\theta }={\left[0,sin\theta ,-cos\theta \right]}^{T}$. The transformation between unit vectors in the spherical coordinates system and the Cartesian coordinate system is defined as:

(3) $$\left[\begin{array}{c} E_{x}\\ E_{z} \end{array}\right]=\left[\begin{array}{cc} -1 & 0\\ 0 & -cos\theta \end{array}\right]\left[\begin{array}{c} E_{\varphi }\\ E_{\theta } \end{array}\right]=\left[\begin{array}{c} -E_{\varphi }\\ -cos\theta E_{\theta } \end{array}\right],$$

The polarization vector p in Cartesian coordinate system of the PSA can be denoted as:

(4) $$p\left(\theta ,\eta ,\gamma \right)=\left[\begin{array}{c} E_{x}\\ E_{z} \end{array}\right]=\left[\begin{array}{c} -cos\gamma \\ -cos\theta sin\gamma e^{j\eta } \end{array}\right],$$

where θ is the grazing angle of the direct signals and $\theta =\theta_{d}$ (Figure 1). The received signal for the PSA under multipath condition can be expressed as:

(5) $$y\left(t\right)=\left(b\left(\theta_{d},\eta ,\gamma \right)+e^{-j2\pi \Delta R/\lambda }\Gamma \left(\psi \right)b\left(\theta_{s},\eta ,\gamma \right)\right)s\left(t\right)+n\left(t\right),$$

where s(t) is the narrow band signal reflected from the target, with the assumption that n(t) is a 2N×1 Gaussian white noise vector with zero mean. Furthermore, $\Delta R=2H_{r}sin\theta_{d}$ is the path difference between the direct and the specular signals; and $b\left(\theta_{d},\eta ,\gamma \right)$, $b\left(\theta_{s},\eta ,\gamma \right)$ are the steering vector of the PSA for the direct and the specular signals, respectively. That can be denoted as:

(6) $$\{\begin{array}{c} b\left(\theta_{d},\eta ,\gamma \right)=a\left(\theta_{d}\right)⨂p\left(\theta_{d},\eta ,\gamma \right)=\left[\begin{array}{c} -a\left(\theta_{d}\right)cos\gamma \\ -a\left(\theta_{d}\right)cos\theta_{d}sin\gamma e^{j\eta } \end{array}\right]\\ b\left(\theta_{s},\eta ,\gamma \right)=a\left(\theta_{s}\right)⨂p\left(\theta_{d},\eta ,\gamma \right)=\left[\begin{array}{c} -a\left(\theta_{s}\right)cos\gamma \\ -a\left(\theta_{s}\right)cos\theta_{d}sin\gamma e^{j\eta } \end{array}\right] \end{array}$$

where ⨂ denote the Kronecker product; and $a\left(\theta_{d}\right)$ and $a\left(\theta_{s}\right)$ are the steering vectors for the direct and the specular signals. These can be expressed as:

(7) $$\{\begin{array}{c} a\left(\theta_{d}\right)={\left[e^{-j\pi \left(N-1\right)dsin\theta_{d}/\lambda },\cdots ,e^{j\pi \left(N-1\right)dsin\theta_{d}/\lambda }\right]}^{T}\\ a\left(\theta_{s}\right)={\left[e^{-j\pi \left(N-1\right)dsin\theta_{s}/\lambda },\cdots ,e^{j\pi \left(N-1\right)dsin\theta_{s}/\lambda }\right]}^{T} \end{array}$$

The Γ(ψ) in Equation (5) is the Fresnel reflection coefficients vector that contains the horizontal and vertical polarization reflection coefficients, which can be denoted as:

(8) $$\Gamma \left(\psi \right)=diag\left(\underset{N}{\underset{︸}{\Gamma_{h}\cdots \Gamma_{h}}},\underset{N}{\underset{︸}{\Gamma_{v}\cdots \Gamma_{v}}}\right)$$

For the horizontal polarization, the electric field is parallel to the interface. For the vertical polarization, the electric field is perpendicular to the interface as defined in a previous study [28]. In this paper, the interface plane is XOY plane as shown in Figure 1. Hence, the electric field in θ direction is called the vertical polarization and the electric field in φ direction is called horizontal polarization. The Fresnel reflection coefficients for horizontal and vertical polarization are defined respectively as follows [28]:

(9) $$\{\begin{array}{c} \Gamma_{h}\left(\psi \right)=\frac{sin\psi -\sqrt{\varepsilon_{c}-cos^{2}\psi }}{sin\psi +\sqrt{\varepsilon_{c}-cos^{2}\psi }},Horizontal\;polarization\;\\ \Gamma_{v}\left(\psi \right)=\frac{\varepsilon_{c}sin\psi -\sqrt{\varepsilon_{c}-cos^{2}\psi }}{\varepsilon_{c}sin\psi +\sqrt{\varepsilon_{c}-cos^{2}\psi }},Vertical\;polarization \end{array}$$

The Fresnel reflection coefficients are determined by the grazing angle ψ and $\varepsilon_{c}$. where $\varepsilon_{c}=\varepsilon_{r}-j60\lambda \sigma$ is the complex permittivity of the smooth ground; $\varepsilon_{r}$ represents the relative permittivity; and σ is the conductivity of the reflective surface. When the grazing angle ψ is close to zero, we can obtain $\Gamma_{h}\left(\psi \right)\approx \Gamma_{h}\left(\psi \right)\approx -1$. In other words, there is very little difference between $\Gamma_{h}\left(\psi \right)$ and $\Gamma_{v}\left(\psi \right)$ when the grazing angle is close to zero. In order to simplify the received signal in Equation (4), we used c to represent the composite vectors, with c denoted as:

(10) $$c=\left(b\left(\theta_{d},\eta ,\gamma \right)+\rho b\left(\theta_{s},\eta ,\gamma \right)\right),$$

where ρ is the fading coefficient vector of the PSA. It can be expressed as:

(11) $$\rho =e^{-j2\pi \Delta R/\lambda }\Gamma \left(\psi \right)=diag\left(\underset{N}{\underset{︸}{\rho_{h}\cdots \rho_{h}}},\underset{N}{\underset{︸}{\rho_{v}\cdots \rho_{v}}}\right),$$

where $\rho_{h}$ and $\rho_{v}$ are the horizontal and vertical polarization fading coefficients, respectively. These are expressed as:

(12) $$\{\begin{array}{c} \rho_{h}=\Gamma_{h}\left(\psi \right)e^{-j2\pi \Delta R/\lambda }=\rho_{h}{}^{\prime }+j\rho_{h}{}^{\prime \prime }\\ \rho_{v}=\Gamma_{v}\left(\psi \right)e^{-j2\pi \Delta R/\lambda }=\rho_{v}{}^{\prime }+j\rho_{v}{}^{\prime \prime } \end{array},$$

where $\rho_{h}{}^{\prime }$ and $\rho_{v}{}^{\prime }$ are the real parts of horizontal and vertical polarization fading coefficients, respectively; and $\rho_{h}{}^{\prime \prime }$ and $\rho_{v}{}^{\prime \prime }$ are the image parts of horizontal and vertical polarization fading coefficients, respectively. The fading coefficients are dependent on the grazing angle ψ, Fresnel reflection coefficient $\Gamma_{h,v}\left(\psi \right)$ and path difference ΔR. By substituting Equation (10) into Equation (5), the received signals in Equation (5) can be simplified as:

(13) y(t)=cs(t)+n(t).

In order to derive the CRB of the elevation angle $\theta_{d}$, we need to calculate the covariance of the received signal. The covariance of the received signal in Equation (13) is:

(14) $$R=\sigma_{s}^{2}cc^{H}+I_{N}\sigma_{n}^{2},$$

where $\sigma_{s}^{2}=E\left[s\left(t\right)s{\left(t\right)}^{H}\right]$ and $\sigma_{n}^{2}=E\left[n\left(t\right)n{\left(t\right)}^{H}\right]$ are the covariance of the signal and noise, respectively. We defined the signal-to-noise ratio of the completely polarized wave signal as: $\mathrm{SNR}=\sigma_{s}^{2}/\sigma_{n}^{2}$. The CRB is a universal lower boundary for the variance of all unbiased estimators of a set of parameters. It is defined as the inverse of the Fisher information matrix (FIM), which describes the amount of information that the data provide about unknown parameters. According to the symmetry geometrical information of the symmetry multipath model as shown in Figure 1, we can obtain $\theta_{s}=-\theta_{d}$. Hence, there are nine unknown parameters $\left\{\theta_{d},\eta ,\gamma ,\rho_{h}{}^{\prime },\rho_{h}{}^{\prime \prime },\rho_{v}{}^{\prime },\rho_{v}{}^{\prime \prime },\sigma_{s}^{2},\sigma_{n}^{2}\right\}$ in the covariance matrix R. The Cramer-Rao Bound (CRB) of the elevation angle $\theta_{d}$ for the PSA can be derived from FIM and the deduction process is included in the Appendix A. The CRB for DOA of grazing angle $\theta_{d}$ is:

(15) $$CRB\left(\theta_{d}\right)={\left[FIM\right]}^{-1}{}_{1,1}.$$

## 3. Polarization Smoothing Generalized MUSIC Algorithm

As shown in Figure 1, the received signals for the PSA under the multipath conditions for the coordinate’s axis can be expressed as follows [17]:

(16) $$\{\begin{array}{c} y_{h}\left(t\right)=y_{x}\left(t\right)=A\left[\begin{array}{c} 1\\ \rho_{h} \end{array}\right]\left(-cos\gamma \right)s\left(t\right)+n\left(t\right)\\ y_{v}\left(t\right)=y_{z}\left(t\right)=A\left[\begin{array}{c} 1\\ \rho_{v} \end{array}\right]\left(-sin\gamma \right)cos\theta_{d}e^{j\eta }s\left(t\right)+n\left(t\right) \end{array},$$

where $A=\left[a\left(\theta_{d}\right),a\left(\theta_{s}\right)\right]$ is the composite steering vector that contains $a\left(\theta_{d}\right)$ and $a\left(\theta_{s}\right)$. In addition, the *X*-direction component corresponds to the horizontal polarization and the *Z*-direction component corresponds to the vertical polarization. The covariance of each polarized components of the received signal in Equation (16) can be expressed as:

(17) $$\{\begin{array}{c} R_{h}=R_{xx}=\sigma_{s}^{2}cos^{2}\gamma A\left[\begin{array}{cc} 1 & \rho_{h}^{*}\\ \rho_{h} & {\left|\rho_{h}\right|}^{2} \end{array}\right]A^{H}+I_{N}\sigma_{n}^{2}\\ R_{v}=R_{zz}=\sigma_{s}^{2}sin^{2}\gamma cos^{2}\theta_{d}A\left[\begin{array}{cc} 1 & \rho_{v}^{*}\\ \rho_{v} & {\left|\rho_{v}\right|}^{2} \end{array}\right]A^{H}+I_{N}\sigma_{n}^{2} \end{array}$$

As we can see from Equation (17), there is only one larger eigenvalue in the covariance matrix $R_{xx}$ and $R_{zz}$ due to the coherence between the direct and the specular signals. The Polarization Smoothing algorithm was proposed by Rahamim [16], which averages the data covariance matrix along the elements in the vector sensors of an array, with the averaging operation performed along the array aperture. The polarization smoothing pre-processing for VHF Polarization Sensitive Array under the multipath conditions can be summarized as:

(18) $$R_{ps}=(R_{h}+\;R_{v})/2\;=\sigma_{s}^{2}AQA^{H}+I_{N}\sigma_{n}^{2}$$

where Q is the averaged envelope covariance matrix of horizontal and vertical polarization subarrays. This can be derived as follows:

(19) $$Q=\frac{cos^{2}\gamma }{2}\left[\begin{array}{cc} 1 & \rho_{h}^{*}\\ \rho_{h} & {\left|\rho_{h}\right|}^{2} \end{array}\right]+\frac{sin^{2}\gamma cos^{2}\theta }{2}\left[\begin{array}{cc} 1 & \rho_{v}^{*}\\ \rho_{v} & {\left|\rho_{v}\right|}^{2} \end{array}\right].$$

As we can see from Equation (19), we can obtain that rank(Q)=2 when $\rho_{h}\neq \rho_{v}$. This indicates that the polarization smoothing pre-processing could eliminate the coherence between the direct and the specular signals. Hence, there are two larger eigenvalues in the polarization smoothing average covariance $R_{ps}$. The decorrelation performance of the polarization smoothing pre-processing is based on the differences of the $\rho_{h}$ and $\rho_{v}$. The PS-MUSIC [17] algorithm is established based on the polarization smoothing technique and MUSIC algorithm for low-angle estimation problem. However, when the grazing angle is close to zero, there is very little difference between $\rho_{h}$ and $\rho_{v}$. Therefore, the decorrelation performance of the polarization smoothing method is reduced when the grazing angle is close to zero. In order to improve the decorrelation performance, the spatial smoothing processing can be exploited after the polarization smoothing pre-processing, which can be used to achieve the PS-SS-MUSIC algorithm. The PS-SS-MUSIC algorithm suffers aperture loss when the number of overlapping subarrays for the spatial smoothing is more than one. In contrast, GMUSIC [27] can be used to solve the low-angle estimation problem, which does not need to solve the coherent problem. Hence, we developed a GMUSIC algorithm for the PSA. 

As we can see from the multipath signal model in Equation (16), the covariance matrices $R_{h}$ and $R_{v}$ can be estimated with L snapshots by $\hat{R_{h}}=\frac{1}{L}\overset{L}{\underset{l=1}{\sum }}y_{h}\left(l\right)y_{h}{\left(l\right)}^{H}$ and $\hat{R_{v}}=\frac{1}{L}\overset{L}{\underset{l=1}{\sum }}y_{v}\left(l\right)y_{v}{\left(l\right)}^{H}$. Furthermore, the sample polarization smoothing average covariance $\hat{R_{ps}}$ can be estimated from Equation (18). The steering vectors $a\left(\theta_{d}\right)$ and $a\left(\theta_{s}\right)$ that correspond to the direct and the specular incoming signals in the signal subspace are therefore orthogonal to the noise subspace $U_{n}$. According to the orthogonality of the signal subspace to the noise subspace, we can derive:

(20) $$\{\begin{array}{c} Pa\left(\theta_{d}\right)=0\\ Pa\left(\theta_{s}\right)=0\\ P=U_{n}U_{n}^{H} \end{array},$$

where P is the projection matrix that is constructed from the eigenvectors $U_{n}$ of the sample covariance matrix $\hat{R_{ps}}$ corresponding to the *N-2* minimum eigenvalue. By constructing the projection matrix $A{\left(\theta_{1},\theta_{2}\right)}^{H}PA\left(\theta_{1},\theta_{2}\right)$, we obtain:

(21) $$A^{H}PA=\left[\begin{array}{cc} a^{H}\left(\theta_{1}\right)Pa\left(\theta_{1}\right) & a^{H}\left(\theta_{1}\right)Pa\left(\theta_{2}\right)\\ a^{H}\left(\theta_{2}\right)Pa\left(\theta_{1}\right) & a^{H}\left(\theta_{2}\right)Pa\left(\theta_{2}\right) \end{array}\right],$$

where $\theta_{1}$ and $\theta_{2}$ are the searching angles; and $A=\left[a\left(\theta_{1}\right),a\left(\theta_{2}\right)\right]$ is the composite steering vector. Obviously, the $A{\left(\theta_{1},\theta_{2}\right)}^{H}PA\left(\theta_{1},\theta_{2}\right)$ is singular if and only if $\left\{\theta_{1}=\theta_{2}\right\},\;\left\{\theta_{1}=\theta_{d}\;\mathrm{and}\;\theta_{2}=\theta_{s}\right\},$ or $\left\{\;\theta_{2}=\theta_{d}\;\mathrm{and}\;\theta_{1}=\theta_{s}\right\}$. According to the geometry information of the symmetry multipath model, we assumed that $\theta_{1}>0^{{}^{\circ}}$ and $\theta_{2}<0^{{}^{\circ}}$ for the range of interest. Therefore, $A{\left(\theta_{1},\theta_{2}\right)}^{H}PA\left(\theta_{1},\theta_{2}\right)$ is singular only if $\theta_{1}=\theta_{d}$, $\theta_{2}=\theta_{s}$, which can obtain that:

(22) $$det\left(A{\left(\theta_{d},\theta_{s}\right)}^{H}PA\left(\theta_{d},\theta_{s}\right)\right)=0,$$

where det() denotes the determinant of the matrix. Moreover, when $\rho_{h}$ is equal to $\rho_{v}$, $A{\left(\theta_{d},\theta_{s}\right)}^{H}PA\left(\theta_{d},\theta_{s}\right)$ also is singular, with the proof included in the Appendix B. After this, the spatial spectrum of PS-GMUSIC is formed by projecting a continuum of composite DOA vector $A\left(\theta_{1},\theta_{2}\right)$ over the given range of interest onto P, with the following form:

(23) $$P_{PS-GMUSIC}\left(\theta_{1},\theta_{2}\right)=\frac{det\left(A{\left(\theta_{1},\theta_{2}\right)}^{H}A\left(\theta_{1},\theta_{2}\right)\right)}{det\left(A{\left(\theta_{1},\theta_{2}\right)}^{H}PA\left(\theta_{1},\theta_{2}\right)\right)},\theta_{1}>0^{{}^{\circ}},\theta_{2}<0^{{}^{\circ}}.$$

The proposed method exploits the composite steering vector $A\left(\theta_{1},\theta_{2}\right)$, making it unnecessary to solve the coherent problem. However, it requires a multidimensional search, which leads to a significant increase in computational burden. Additionally, based on the geometry of symmetry multipath model (Figure 1), it can obtain the following expression for the searching angles:

(24) $$\theta_{2}=-\theta_{1}.$$

Substituting Equation (24) into Equation (23), the two-dimensional angle searching in Equation (23) can be transformed into one-dimensional angle searching, which is:

(25) $$P_{PS-GMUSIC}\left(\theta_{1}\right)=\frac{det\left(A{\left(\theta_{1},-\theta_{1}\right)}^{H}A\left(\theta_{1},-\theta_{1}\right)\right)}{det\left(A{\left(\theta_{1},-\theta_{1}\right)}^{H}PA\left(\theta_{1},-\theta_{1}\right)\right)},\theta_{1}>0^{{}^{\circ}}$$

Up to now, we have achieved the PS-GMUSIC algorithm. The procedure of the proposed method can be summarized as follows:

1. Construct sample covariance matrices $\hat{R_{h}}$ and $\hat{R_{v}}$;
2. Use the polarization smoothing pre-processing to get $\hat{R_{ps}}$;
3. Compute the noise subspace $U_{n}$ and Projection matrix P by means of EVD (Eigenvalue Decomposition) using $\hat{R_{ps}}$; and
4. Establish the PS-GMUSIC spatial spectrum based on Equation (25).

The geometry information of the multipath model can be exploited to reduce the computational cost for the PS-GMUSIC algorithm. After the transformation from the two-dimensional searching into the one-dimensional searching, the computation burden of the PS-GMUSIC is $O_{PS-GMUSIC}\left(N^{3}+2LN^{2}+n\left(N^{3}+6N^{2}+20N+4\right)\right)$, where L is the number of the snapshots and n is the number of the searching angles. 

## 4. Results

In this section, some simulation results are presented to assess the performance of the proposed method in terms of CRB, RMSE and Pseudo spectra. We adopted the VHF PSA radar system with $N=10,f_{0}=300\;\mathrm{MHZ}$, $H_{r}=10\;m,SNR=10\;\mathrm{dB},d=\lambda /2$ and L=256. We assumed that there is one point target over the calm seawater $\left(\varepsilon_{r}=80,\sigma =4\;s/m\right)$ for the following simulations.

### 4.1. CRB Analysis

**Figure 2:**
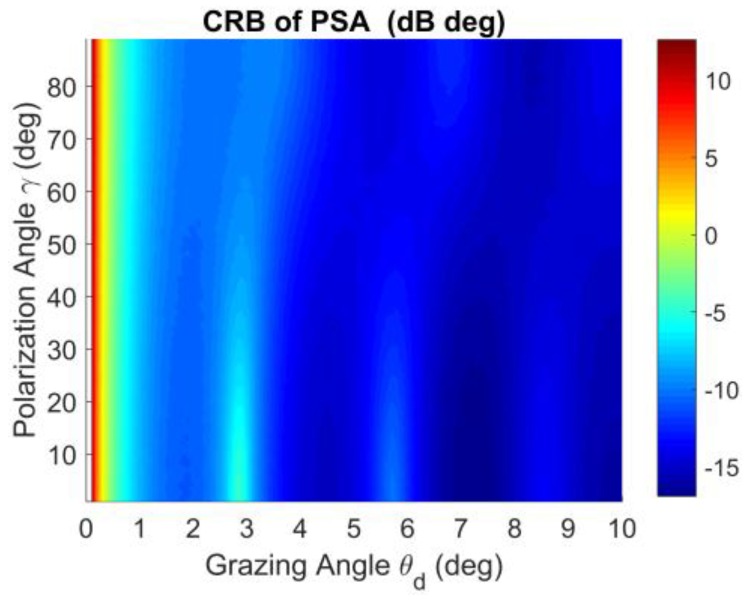
CRB with respect to polarization angle γ and DOA of grazing angle $\theta_{d}$.

The CRB of the PSA VHF radar system for different grazing angles and polarization angles is shown in Figure 2. The polarization angle γ lies in $\left(0^{{}^{\circ}}–90^{{}^{\circ}}\right)$. The grazing angle of the target varies from $0^{{}^{\circ}}$ to $10^{{}^{\circ}}$. The CRB is very high when the grazing angle is close to zero. That is still a challenging problem for low-angle estimation. There are some periodic peaks and troughs for the CRB when the polarization angle γ lies in $\left(0^{{}^{\circ}}–20^{{}^{\circ}}\right)$ and $\left(70^{{}^{\circ}}–90^{{}^{\circ}}\right)$, which is shown in Figure 2. When γ lies in the interval of $\left[30^{{}^{\circ}}–60^{{}^{\circ}}\right]$, the CRB is lower at the rings for the different grazing angles. This is reasonable since the energy of horizontal polarization and vertical polarization component is almost the same when γ lies in the interval of $\left[30^{{}^{\circ}}–60^{{}^{\circ}}\right]$. However, when γ lies in $\left(0^{{}^{\circ}}–20^{{}^{\circ}}\right)$, the horizontal polarization component dominates, while the vertical polarization component dominates when γ lies in $\left(70^{{}^{\circ}}–90^{{}^{\circ}}\right)$. Therefore, the polarization angle $\gamma \in \left[30^{{}^{\circ}}–60^{{}^{\circ}}\right]$ is recommended for the PSA.

**Figure 3:**
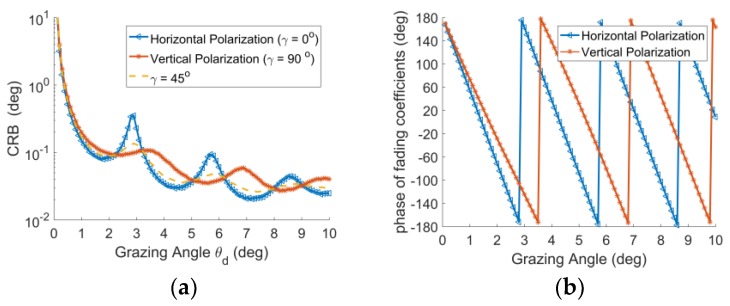
(**a**) CRB with respect to DOA of grazing angle; and (**b**) Phase of fading coefficients with respect to DOA of grazing angle.

As we can see from Figure 3, the CRBs of horizontal and vertical polarization have ‘singular points’ (Peak points) when the phase of fading coefficients is $\pm 180^{{}^{\circ}}$. For example, the grazing angle is $2.9^{{}^{\circ}},3.5^{{}^{\circ}},5.7^{{}^{\circ}}\;\mathrm{and}\;6.8^{{}^{\circ}},$ which is shown in Figure 3a,b. It is because the SNR is reduced when the phase of fading coefficients is $\pm 180^{{}^{\circ}}$ due to the cancellation between the direct and reflected signals. This is why the performance of GMUSIC algorithm corrupts when the phase of fading coefficients is $\pm 180^{{}^{\circ}}$ with the traditional phase array. In contrast, the CRB of PSA could merge the ‘singular points’ for $\gamma =45^{{}^{\circ}}$ based on the different phases of fading coefficients for the horizontal and vertical polarization components in the PSA.

### 4.2. RMSE of the Proposed Method

In order to assess the angle estimation performance of the proposed algorithm, the Monte Carlo simulations are presented in this part. The root-mean-square error (RMSE) is defined below:

$$\delta_{RMSE}=\sqrt{\frac{1}{M}\sum_{i=1}^{M}{\left|{\hat{\theta }}_{i,d}-\theta_{d}\right|}^{2}}$$

where ${\hat{\theta }}_{i,d}$ is the $i_{th}$ angle estimation of the DOA of grazing angle $\theta_{d}$, M is the number of Monte Carlo simulations. Assume M=1000 for the following simulations. The RMSE of the proposed method was compared with PS-MUSIC [17], PS-SS-MUSIC [16] and IBMUSIC [29] algorithms. The Spatial Smoothing utilizes the forward and backward smoothing [23] with two overlapping subarrays in the simulation. The iteration beamspace multiple signal classification (IBMUSIC) algorithm was proposed in [29] to realize unbiased estimation and reduce the calculation time for DOA of CDMA Multipath signals. This approach can be utilized for low angle estimation performance with PSA. The performance of the IBMUSIC algorithm heavily depends on initial value [29]. And assume the initial value is 1° for the following simulations. The beam number of beamspace is 5 for IBMUSIC algorithm and assume the deviation precision value as 0.001 for iteration.

**Figure 4:**
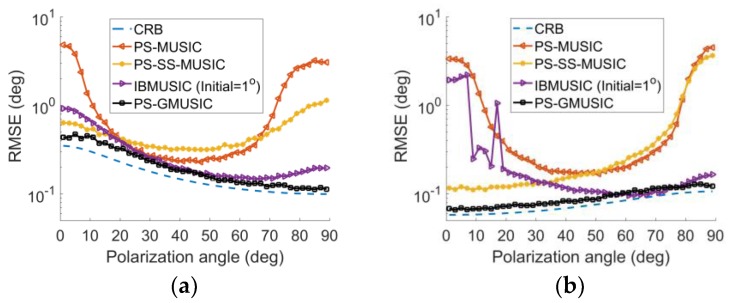
RMSE with respect to the polarization angle γ for SNR=10 dB for: (**a**) DOA of $\theta_{d}=2.9^{{}^{\circ}}$; (**b**) DOA of $\theta_{d}$ = $3.5^{{}^{\circ}}$.

Figure 4a,b show the RMSE with respect to the polarization angle γ. We assumed that the direct signal arrived at an angle of 2.9° and 3.5° when the phase of fading coefficients is $\pm 180^{{}^{\circ}}$ (Figure 3). Since the decorrelation performance of the polarization smoothing are determined by the polarization angle [17], the RMSE of PS-MUSIC varies according to the polarization angle as shown in Figure 4. When γ lies in $\left(0^{{}^{\circ}}–20^{{}^{\circ}}\right)$, the horizontal polarization component dominates, while the vertical polarization component dominates when γ lies in $\left(70^{{}^{\circ}}–90^{{}^{\circ}}\right)$.And the decorrelation of the polarization smoothing is poor when the vertical polarization or horizontal polarization component dominate, hence the performance of PS-MUSIC is nearly destroyed when γ lies in $\left(70^{{}^{\circ}}–90^{{}^{\circ}}\right)$ and $\left(0^{{}^{\circ}}–20^{{}^{\circ}}\right)$, as shown in Figure 4. The PS-SS-MUSIC algorithm takes advantage the polarization smoothing method and spatial smoothing approach at the same time to improve the decorrelation performance. Hence, the performance of PS-SS-MUSIC algorithm is much better than PS-MUSIC when γ lies in $\left(70^{{}^{\circ}}–90^{{}^{\circ}}\right)$ and $\left(0^{{}^{\circ}}–20^{{}^{\circ}}\right)$ as shown in Figure 4a. When γ lies in $\left[20^{{}^{\circ}}–70^{{}^{\circ}}\right]$ in Figure 4a or $\left[50^{{}^{\circ}}–70^{{}^{\circ}}\right]$ in Figure 4b, the RMSE of PS-SS-MUSIC is higher than those of the PS-MUSIC algorithm due to the aperture loss. The performance of IBMUSIC algorithm is poor when γ lies in $\left(70^{{}^{\circ}}–90^{{}^{\circ}}\right)$ and $\left(0^{{}^{\circ}}–20^{{}^{\circ}}\right)$ as shown in Figure 4a. It because the decorrelation of the polarization smoothing is poor when the single polarized components dominate. Unlike the other schemes, when the single polarized components dominate the proposed method does not need to solve the coherence between the direct signal and specular signals. Hence, our proposed scheme retains more stable performance for all the polarization angles as shown in Figure 4. The RMSE of the proposed method is much higher when γ lies in $\left(0^{{}^{\circ}}–20^{{}^{\circ}}\right)$ in Figure 4a or $\left(80^{{}^{\circ}}–90^{{}^{\circ}}\right)$ in Figure 4b. This is because the SNR is reduced when the phase of fading coefficients is $\pm 180^{{}^{\circ}}$ for single polarized dominates. To achieve more stable angle estimation performance for all DOA of grazing angle, $\gamma =45^{{}^{\circ}}$ is assumed in the following simulations.

**Figure 5:**
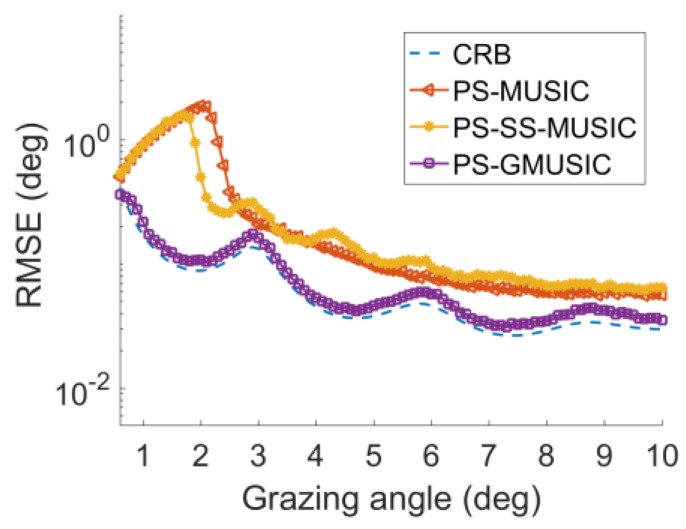
RMSE with respect to DOA of grazing angle for $\gamma =45^{{}^{\circ}},SNR=10\;\mathrm{dB}$.

Figure 5 demonstrates the RMSE with respect to the DOA of grazing angle $\theta_{d}$. From Figure 5, the proposed method has better performance than PS-MUSIC and PS-SS-MUSIC. The proposed method does not have any antenna aperture loss in comparison with PS-SS-MUSIC. The RMSE of the PS-MUSIC and PS-SS-MUSIC algorithm are corrupted when the elevation angle is less than $2.5^{{}^{\circ}}$ (Figure 5). The RMSE of the proposed method still is consistent with the CRB curve, even when the grazing angle is around $0.5^{{}^{\circ}}$. That is because the decorrelation of polarization smoothing is poor for polarization smoothing when the grazing angle is close to zero. While the proposed method doesn’t need to solve the coherent problem. Hence the better performance is expected.

**Figure 6:**
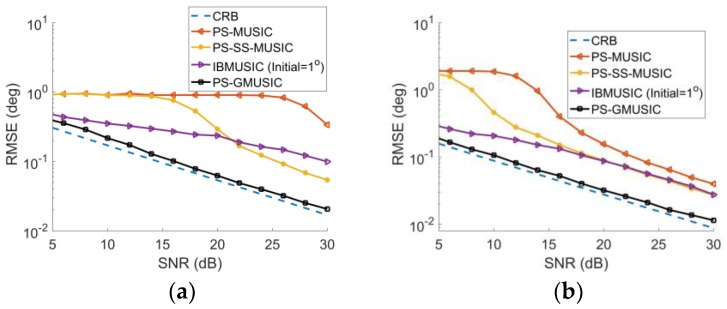
RMSE with respect to SNR for $\gamma =45^{{}^{\circ}}$ with: (**a**) DOA of $\theta_{d}=1^{{}^{\circ}}$; and (**b**) DOA of $\theta_{d}=2^{{}^{\circ}}$.

Figure 6a,b represent the RMSE with respect to SNR with DOA of $\theta_{d}$ is 1° and 2° which are close to zero. As expected, the angular accuracy is improved with an increase in SNR. The proposed method has the lowest RMSE of angle estimation for different SNR in comparison with PS-MUSIC, PS-SS-MUSIC and IBMUSIC algorithms (Figure 6). Under the low-SNR condition, the PS-MUSIC and PS-SS-MUSIC corrupts (Figure 6). However, the proposed method still is consistent with the CRB curve, even in the low SNR conditions.

### 4.3. Pseudo Spectra of the Proposed Method

**Figure 7:**
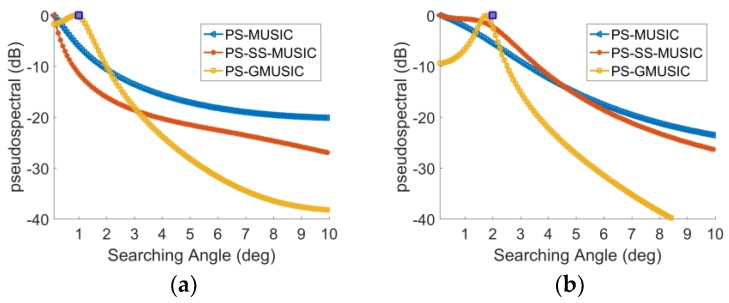
Pseudo spectra of different methods with $\gamma =45^{{}^{\circ}},SNR=10\;\mathrm{dB}$. (**a**) $\theta_{d}=1^{{}^{\circ}}$; (**b**) $\theta_{d}=2^{{}^{\circ}}$.

As we can see from Figure 7, when the DOA of grazing angle $\theta_{d}$ is close to zero, the pseudo spectra of PS-MUSIC and SS-PS-MUSIC algorithm do not have obvious peaks, while the PS-GMUSIC algorithm still has a peak. That indicates that the proposed method has better angle resolution and can achieve better low-angle estimation performance when the grazing angle is close to zero.

## 5. Conclusions

The PS-GMUSIC method for low-angle estimation with PSA radar system is proposed in this paper. The proposed algorithm takes advantage of the polarization smoothing method and the generalized MUSIC algorithm to achieve better low-angle estimation performance. The PS-GMUSIC algorithm utilizes the composite steering vector instead of the ordinary steering vector, which does not need to solve the coherent problem. Meanwhile, the information of the symmetry geometry of multipath model is exploited to reduce the computational burden for the proposed algorithm. The PS-GMUSIC has a more stable performance for all polarization angles. In addition, the proposed method has better angle estimation performance in low-angle area in comparison with PS-MUSIC, PS-SS-MUSIC and IBMUSIC algorithms, especially when the grazing angle is close to zero. The strategy that proposed in this paper also can be exploited in the MIMO radar system for the low-angle estimation problem.

## Appendix A

The CRB for PSA radar system is derived in this part. Fisher information Matrix (FIM) is the foundation of the Cramer-Rao Bound. The FIM of the multipath model in this paper can be denoted as:

(A1) $$FIM=\left[F_{u_{i}u_{j}}\right].$$

where $F_{u_{i}u_{j}}$ is the (i,j)th element of the FIM, which is defined as:

(A2) $$F_{u_{i}u_{j}}=L\cdot Tr\left(R^{-1}\frac{\partial R}{\partial u_{i}}R^{-1}\frac{\partial R}{\partial u_{j}}\right)=F_{u_{j}u_{i}}.$$

There are nine unknown parameters $\left\{\theta_{d},\eta ,\gamma ,\rho_{h}{}^{\prime },\rho_{h}{}^{\prime \prime },\rho_{v}{}^{\prime },\rho_{v}{}^{\prime \prime },\sigma_{s}^{2},\sigma_{n}^{2}\right\}$ in the covariance matrix R. and $u_{i,j}\in \left\{\theta_{d},\eta ,\gamma ,\rho_{h}{}^{\prime },\rho_{h}{}^{\prime \prime },\rho_{v}{}^{\prime },\rho_{v}{}^{\prime \prime },\sigma_{s}^{2},\sigma_{n}^{2}\right\}$, where L is number of the snapshots of the received signal. When $u_{i},u_{j}\in \left\{\sigma_{s}^{2},\sigma_{n}^{2}\right\}$ $F_{u_{i}u_{j}}$ can be simplified as:

(A3) $$\{\begin{array}{c} F_{\sigma_{s}^{2},\sigma_{s}^{2}}=L\cdot \left(c^{H}R^{-1}cc^{H}R^{-1}c\right)\\ F_{\sigma_{n}^{2},\sigma_{n}^{2}}=L\cdot Tr\left(R^{-1}R^{-1}\right) \end{array}$$

When $u_{i},u_{j}\in \left\{\theta_{d},\eta ,\gamma ,\rho_{h}{}^{\prime },\rho_{h}{}^{\prime \prime },\rho_{v}{}^{\prime },\rho_{v}{}^{\prime \prime }\right\}$, $F_{u_{i}u_{j}}$ can be simplified as:

(A4) $$F_{u_{i}u_{j}}=2\sigma_{s}^{4}\cdot L\cdot Re\left(c^{H}R^{-1}\frac{\partial c}{\partial u_{i}}c^{H}R^{-1}\frac{\partial c}{\partial u_{j}}+c^{H}R^{-1}c\frac{\partial c^{H}}{\partial u_{i}}R^{-1}\frac{\partial c}{\partial u_{j}}\right),$$

where c is the composite steering vector of the received signal for PSA; and $\sigma_{s}^{2}$ is the variance of the signal. When $u_{i}\in \left\{\sigma_{s}^{2},\sigma_{n}^{2}\right\},u_{j}\in \left\{\theta_{d},\eta ,\gamma ,\rho_{h}{}^{\prime },\rho_{h}{}^{\prime \prime },\rho_{v}{}^{\prime },\rho_{v}{}^{\prime \prime }\right\}$, $F_{u_{i}u_{j}}$ can be simplified as:

(A5) $$\{\begin{array}{c} F_{\sigma_{s}^{2}u_{j}}=2\sigma_{s}^{2}\cdot L\cdot Re\left(c^{H}R^{-1}cc^{H}R^{-1}\frac{\partial c}{\partial u_{j}}\right)\\ F_{\sigma_{s}^{2},\sigma_{n}^{2}}=L\cdot Tr\left(R^{-1}cc^{H}R^{-1}\right)\\ F_{\sigma_{n}^{2}u_{j}}=2\sigma_{s}^{2}\cdot L\cdot Re\left(c^{H}R^{-1}R^{-1}\frac{\partial c}{\partial u_{j}}\right)\\ F_{\sigma_{n}^{2},\sigma_{s}^{2}}=L\cdot Tr\left(R^{-1}R^{-1}cc^{H}\right) \end{array}.$$

In order to establish the FIM, we only need to know the partial derivatives of composite steering vector c with respect to $u_{i}\in \left\{\theta_{d},\eta ,\gamma ,\rho_{h}{}^{\prime },\rho_{h}{}^{\prime \prime },\rho_{v}{}^{\prime },\rho_{v}{}^{\prime \prime }\right\}$. According to the definition of the composite steering vector c in Equation (9), the partial derivatives can be derived as:

(A6) $$\{\begin{array}{c} \frac{\partial c}{\partial \theta_{d}}=\frac{\partial b\left(\theta_{d},\eta ,\gamma \right)}{\partial \theta_{d}}+\rho \frac{\partial b\left(-\theta_{d},\eta ,\gamma \right)}{\partial \theta_{d}}\\ \frac{\partial c}{\partial \eta }=\frac{\partial b\left(\theta_{d},\eta ,\gamma \right)}{\partial \eta }+\rho \frac{\partial b\left(-\theta_{d},\eta ,\gamma \right)}{\partial \eta }\\ \frac{\partial c}{\partial \gamma }=\frac{\partial b\left(\theta_{d},\eta ,\gamma \right)}{\partial \gamma }+\rho \frac{\partial b\left(-\theta_{d},\eta ,\gamma \right)}{\partial \gamma } \end{array}$$

(A7) $$\{\begin{array}{c} \frac{\partial c}{\partial \rho_{h}{}^{\prime }}=diag\left(\underset{N}{\underset{︸}{1\cdots 1}},\underset{N}{\underset{︸}{0\cdots 0}}\right)b\left(-\theta_{d},\eta ,\gamma \right)\\ \frac{\partial c}{\partial \rho_{h}{}^{\prime \prime }}=diag\left(\underset{N}{\underset{︸}{j\cdots j}},\underset{N}{\underset{︸}{0\cdots 0}}\right)b\left(-\theta_{d},\eta ,\gamma \right)\\ \frac{\partial c}{\partial \rho_{v}{}^{\prime }}=diag\left(\underset{N}{\underset{︸}{0\cdots 0}},\underset{N}{\underset{︸}{1\cdots 1}}\right)b\left(-\theta_{d},\eta ,\gamma \right)\\ \frac{\partial c}{\partial \rho_{v}{}^{\prime \prime }}=diag\left(\underset{N}{\underset{︸}{0\cdots 0}},\underset{N}{\underset{︸}{j\cdots j}}\right)b\left(-\theta_{d},\eta ,\gamma \right) \end{array},$$

where:

(A8) $$\{\begin{array}{c} \frac{\partial b\left(\theta_{d},\eta ,\gamma \right)}{\partial \theta_{d}}=\left[\begin{array}{c} -d_{d}⊙a\left(\theta_{d}\right)cos\gamma \\ -d_{d}⊙a\left(\theta_{d}\right)cos\theta_{d}sin\gamma e^{j\eta_{1}}+a\left(\theta_{d}\right)sin\theta_{d}sin\gamma e^{j\eta } \end{array}\right]\\ \frac{\partial b\left(-\theta_{d},\eta ,\gamma \right)}{\partial \theta_{d}}=\left[\begin{array}{c} d_{d}⊙a\left(-\theta_{d}\right)cos\gamma \\ d_{d}⊙a\left(-\theta_{d}\right)cos\theta_{d}sin\gamma e^{j\eta_{1}}-a\left(-\theta_{d}\right)sin\theta_{d}sin\gamma e^{j\eta } \end{array}\right] \end{array}$$

(A9) $$\{\begin{array}{c} \frac{\partial b\left(\theta_{d},\eta ,\gamma \right)}{\partial \eta }=\left[\begin{array}{c} 0\\ -ja\left(\theta_{d}\right)cos\theta_{d}sin\gamma e^{j\eta } \end{array}\right]\\ \frac{\partial b\left(-\theta_{d},\eta ,\gamma \right)}{\partial \eta }=\left[\begin{array}{c} 0\\ -ja\left(-\theta_{d}\right)cos\theta_{d}sin\gamma e^{j\eta } \end{array}\right] \end{array}.$$

(A10) $$\{\begin{array}{c} \frac{\partial b\left(\theta_{d},\eta ,\gamma \right)}{\partial \gamma }=\left[\begin{array}{c} a\left(\theta_{d}\right)sin\gamma \\ -a\left(\theta_{d}\right)cos\theta_{d}cos\gamma e^{j\eta } \end{array}\right]\\ \frac{\partial b\left(-\theta_{d},\eta ,\gamma \right)}{\partial \gamma }=\left[\begin{array}{c} a\left(-\theta_{d}\right)sin\gamma \\ -a\left(-\theta_{d}\right)cos\theta_{d}cos\gamma e^{j\eta } \end{array}\right] \end{array}$$

(A11) $$d_{d}=j\pi \cos\theta_{d}{\left[-\frac{N-1}{2},-\frac{N-2}{2},\cdots ,\frac{N-2}{2},\frac{N-1}{2}\right]}^{T}$$

where ⨀ represents the element-wise product. By substituting (A6)–(A11) into (A3)–(A5) to establish the Fisher Information Matrix FIM, the CRB of the $\theta_{d}$ can be subsequently calculated as:

(A12) $$CRB\left(\theta_{d}\right)={\left[FIM\right]}^{-1}{}_{1,1}.$$

## Appendix B

When $\rho_{h}$ is equal to $\rho_{v}$, the envelope matrix Q can be simplified as:

(A13) $$Q=\frac{cos^{2}\gamma }{2}\left[\begin{array}{cc} 1 & \rho_{h}^{*}\\ \rho_{h} & {\left|\rho_{h}\right|}^{2} \end{array}\right]+\frac{sin^{2}\gamma cos^{2}\theta }{2}\left[\begin{array}{cc} 1 & \rho_{v}^{*}\\ \rho_{v} & {\left|\rho_{v}\right|}^{2} \end{array}\right]=\frac{cos^{2}\gamma +sin^{2}\gamma cos^{2}\theta_{d}}{2}\beta \beta^{H},$$

where:

(A14) $$\beta =\left[\begin{array}{c} 1\\ \rho_{h} \end{array}\right].$$

As we can see from (A13), we can obtain rank(Q)=1. Hence, the polarization smoothing pre-processing cannot eliminate the coherence between the direct and reflected signal when $\rho_{h}$ is equal to $\rho_{v}$. By substituting (A13) into Equation (5), the covariance of the received signal after the polarization smoothing can be expressed as:

(A15) $$R_{ps}=\sigma_{s}^{2}\frac{cos^{2}\gamma +sin^{2}\gamma cos^{2}\theta_{d}}{2}A\beta {\left(A\beta \right)}^{H}+I_{N}\sigma_{n}^{2}=\sigma_{s}^{2}AQA^{H}+I_{N}\sigma_{n}^{2}.$$

Because the rank of Q is one, there is only one larger eigenvalue in the Eigen-decomposition of the average covariance matrix $R_{ps}$. The eigen-decomposition of $R_{ps}$ can be expressed as:

(A16) $$R_{ps}=U_{s}E_{s}U_{s}^{H}+U_{n}E_{n}U_{n}^{H}$$

where $E_{s}=diag\left(\sigma_{1}\right)$ and $\sigma_{1}$ is the largest eigenvalues of $R_{ps}$; $U_{s}$ is the eigenvector corresponding to the largest eigenvalue $\sigma_{1}$, which spans the signal subspace; $E_{n}=diag\left(\sigma_{2},\cdots ,\sigma_{N}\right)$ is a diagnosis matrix constructed by the N−1 minimum eigenvalue of $R_{ps}$; and $U_{n}$ contains the N−1 eigenvectors corresponding to the N−1 minimum eigenvalue of $R_{ps}$, which span the noise subspace. We can obtain that:

(A17) $$\{\begin{array}{c} PA\left(\theta_{d},\theta_{s}\right)\beta =0\\ P=U_{n}U_{n}^{H} \end{array},$$

where $A\left(\theta_{d},\theta_{s}\right)=\left[a\left(\theta_{d}\right),a\left(\theta_{s}\right)\right]$ and P is the projection matrix that is constructed from the eigenvectors $U_{n}$. According to (A17), we can obtain that:

(A18) $$\{\begin{array}{c} Pa\left(\theta_{d}\right)=-\rho_{h}Pa\left(\theta_{s}\right)\\ a^{H}\left(\theta_{d}\right)P=-\rho_{h}^{*}a^{H}\left(\theta_{s}\right)P \end{array}$$

We constructed the projection matrix $A{\left(\theta_{d},\theta_{s}\right)}^{H}PA\left(\theta_{d},\theta_{s}\right)$ as follows:

(A19) $$A^{H}PA=\left[\begin{array}{cc} a^{H}\left(\theta_{d}\right)Pa\left(\theta_{d}\right) & a^{H}\left(\theta_{d}\right)Pa\left(\theta_{s}\right)\\ a^{H}\left(\theta_{s}\right)Pa\left(\theta_{d}\right) & a^{H}\left(\theta_{s}\right)Pa\left(\theta_{s}\right) \end{array}\right].$$

By substituting (A18) into (A19), (A19) can be simplified as:

(A20) $$A^{H}PA=\left[\begin{array}{cc} \rho_{h}\rho_{h}^{*} & -\rho_{h}^{*}\\ -\rho_{h} & 1 \end{array}\right]a^{H}\left(\theta_{s}\right)Pa\left(\theta_{s}\right).$$

Obviously, the $A{\left(\theta_{d},\theta_{s}\right)}^{H}PA\left(\theta_{d},\theta_{s}\right)$ is singular and hence:

(A21) $$det\left(A{\left(\theta_{d},\theta_{s}\right)}^{H}PA\left(\theta_{d},\theta_{s}\right)\right)=0.$$

When $\rho_{h}=\rho_{v}$, $A{\left(\theta_{d},\theta_{s}\right)}^{H}PA\left(\theta_{d},\theta_{s}\right)$ is also singular.
